# Health Effects of Airborne Exposures from Concentrated Animal Feeding Operations

**DOI:** 10.1289/ehp.8835

**Published:** 2006-11-14

**Authors:** Dick Heederik, Torben Sigsgaard, Peter S. Thorne, Joel N. Kline, Rachel Avery, Jakob H. Bønløkke, Elizabeth A. Chrischilles, James A. Dosman, Caroline Duchaine, Steven R. Kirkhorn, Katarina Kulhankova, James A. Merchant

**Affiliations:** 1 University of Utrecht, Utrecht, the Netherlands; 2 University of Aarhus, Aarhus, Denmark; 3 The University of Iowa, Iowa City, Iowa, USA; 4 University of North Carolina, Chapel Hill, North Carolina, USA; 5 University of Saskatchewan, Saskatoon, Saskatchewan, Canada; 6 Laval University, Québec City, Québec, Canada; 7 National Farm Medicine Center, Marshfield, Wisconsin, USA

**Keywords:** air quality, asthma, biological agents, endotoxin, inflammation, odor, poultry, swine

## Abstract

Toxic gases, vapors, and particles are emitted from concentrated animal feeding operations (CAFOs) into the general environment. These include ammonia, hydrogen sulfide, carbon dioxide, malodorous vapors, and particles contaminated with a wide range of microorganisms. Little is known about the health risks of exposure to these agents for people living in the surrounding areas. Malodor is one of the predominant concerns, and there is evidence that psychophysiologic changes may occur as a result of exposure to malodorous compounds. There is a paucity of data regarding community adverse health effects related to low-level gas and particulate emissions. Most information comes from studies among workers in CAFO installations. Research over the last decades has shown that microbial exposures, especially endotoxin exposure, are related to deleterious respiratory health effects, of which cross-shift lung function decline and accelerated decline over time are the most pronounced effects. Studies in naïve subjects and workers have shown respiratory inflammatory responses related to the microbial load. This working group, which was part of the Conference on Environmental Health Impacts of Concentrated Animal Feeding Operations: Anticipating Hazards—Searching for Solutions, concluded that there is a great need to evaluate health effects from exposures to the toxic gases, vapors, and particles emitted into the general environment by CAFOs. Research should focus not only on nuisance and odors but also on potential health effects from microbial exposures, concentrating on susceptible subgroups, especially asthmatic children and the elderly, since these exposures have been shown to be related to respiratory health effects among workers in CAFOs.

## Background and Recent Developments

### Gases and vapors

A number of toxic gases and vapors are emitted by concentrated animal feeding operations (CAFOs) into the work and general environments. In particular, occupational studies have yielded information about exposure levels of ammonia (NH_3_), hydrogen sulfide (H_2_S), and carbon dioxide (CO_2_). The characteristic odor of a CAFO is the result of a complex mixture of these gases and many volatile and semivolatile organic compounds. Odor emissions are especially associated with quality of life issues for exposed populations. Specific gases such as H_2_S are being used as proxies to estimate or regulate exposure to the whole complex mixture. Although this approach has the advantage of simplicity and exposure estimates can be compared with guideline exposure values, it also has several limitations. Situations may arise where the surrogate compound does not co-vary with other toxicants in the mixture. The issue of which specific community health effects may result from CAFO emissions is open and controversial. There is limited evidence that symptom patterns may be the result of CAFO exposures in individuals living in their vicinity. Changes in immunoglobulin A responses have been observed in individuals and associated with exposure to odor, suggesting that psychophysiologic responses can occur (Avery et al. 2004). The underlying mechanistic explanation is that these physiologic changes are most likely stress related; however, other mechanisms including sensitization may also contribute.

Very little information exists about lung function changes among populations living in the vicinity of CAFOs. Emission studies, in combination with modeling approaches, are helpful but not sufficient to relate exposures to health effects because the exposure depends on personal activity patterns and time spent near different sources. A recent report from Germany found that people residing in proximity to many CAFOs (> 12 within a 500-m radius) experienced significantly increased prevalence of self-reported wheezing and decreased forced expiratory volume in 1 sec (FEV_1_) indicative of inflammatory effects of CAFO emissions in the lungs (Radon et al. 2005). However, a problem with the interpretation is that clear differences existed in sensitization rates between rural participants and the urban comparison population. In addition, it can be expected that differences existed within the rural population associated with childhood exposure patterns to animals on these farms compared with those experienced with CAFOs. This raises methodologic issues regarding appropriate comparison populations and confounders or effect-modifying variables that need to be included in multiple regression models to make accurate comparisons. Nevertheless, the results from this study are of interest and similar studies need to be undertaken in other populations with more subjects living in proximity to CAFOs.

Most information on potential health effects comes from working populations. Gases seem to play a limited role in the explanation of work-related respiratory symptoms in CAFO workers, but this may not be true for humans living in the surrounding areas. The distribution of adverse effects by age is different, and susceptibility may be an important issue, with children and elderly individuals belonging to the most vulnerable populations. Also, socioeconomic relationships between workers and companies, as opposed to neighbors and companies, are different, and this will have an impact on the willingness to tolerate hazardous exposures, and bear an increased burden of ill health.

### Exposure inside CAFOs

In early studies on respiratory health of CAFO workers, several constituents of dust have been considered. Exposure to allergens from pigs and storage mites has received some attention, but most studies have shown that sensitization rates to swine urine proteins among farmers are relatively low and cannot explain the high symptom rates in CAFO workers (Brouwer et al. 1990; Cormier et al. 1991; Crook et al. 1991; Harries and Cromwell 1982; Katila et al. 1981). Similarly, responses against storage mites seem to be explained mainly by cross-reactivity to house dust mites.

Early studies have shown that the CAFO industry environment is rich in microbial life. Kiekhaefer et al. (1995) made detailed microbial characterizations of the environment in various types of CAFOs across seasons and showed that moderately elevated culturable mold levels exist with an overall mean concentration of 5.3 × 10^3^ colony-forming units/m^3^ and mean total organisms (orgs) concentrations of 1.1 × 10^8^ orgs/m^3^. The predominant fungal types were *Cladosporium* and *Alternaria* in the summer and fall and yeasts *Penicillium* and *Fusarium* in the winter and spring. Mainly gram-positive bacteria are found in CAFOs, but gram negatives play an important role as well. As a result, endotoxin levels are clearly elevated. Although microbial markers other than endotoxin have been associated with respiratory health and specific respiratory inflammation (Zhiping et al. 1996), endotoxin has been most extensively studied in experimental studies with naïve subjects and epidemiologic studies using a range of study designs.

### Endotoxin exposure

Particulate exposure in the environs surrounding CAFOs occurs but has not received much attention to date. Considerable information exists about work-place exposure to dust from CAFOs (Schenker et al. 1998), and since the 1980s, endotoxins have been identified as important causal and toxic agents (Attwood et al. 1987; Donham et al. 1984).

Endotoxin is composed of lipopolysaccharides (LPS) and is a nonallergenic cell-wall component of gram-negative bacteria with strong proinflammatory properties. Endotoxins are ubiquitous and commonly present inside CAFOs, the general environment, and house dust (Douwes et al. 1998, 2000; Michel et al. 1991; Peterson et al. 1964; Schenker et al. 1998; Thorne et al. 2005). Very high endotoxin exposure occurs in farming, particularly livestock farming (Kullman et al. 1998; Schenker et al. 1998). Elevated endotoxin levels are present in homes where children have regular contact with farm animals and in homes where pets are present (Douwes et al. 2000; Thorne et al. 2003; von Mutius et al. 2000). Larger numbers of family members, poverty, and roach infestation are also associated with higher endotoxin in homes (Thorne et al. 2003, 2005). Inhalatory endotoxin exposure has been associated with a range of respiratory health effects in both the work and the domestic environment (Douwes and Heederik 1997; Douwes et al. 2002). Because results of research on respiratory health effects of endotoxin exposure were focused on different aspects for the occupational and domestic environments, overall results are reviewed briefly here for both environments.

### Work environment exposures and adult onset allergy and asthma

Early studies on endotoxin exposure and farming activities began in about 1982. Swine production facilities have especially been studied since that period. Endotoxin exposure is high for workers in these industries, and several large-scale studies suggest that the mean exposure varies between a few hundred up to 15,000 endotoxin units (EU)/m^3^ of air in situations where ventilation is limited because of extreme climatic conditions (Douwes and Heederik 1997). These exposures have been associated with increased symptoms, both respiratory and systemic (Rylander et al. 1989), across-shift lung function changes (Donham et al. 2000), reduced lung function in cross-sectional studies and accelerated decline in lung function in longitudinal studies (Vogelzang et al. 1998). Neutrophil-mediated inflammation has been observed in naïve subjects and swine confinement workers after exposure that is limited to periods of a few hours (Jagielo et al. 1996; Sandström et al. 1992; Von Essen and Romberger 2003). The exact pathophysiology is not clear, but it is well established that it is mediated by an acute inflammatory response involving a variety of cytokines, including interleukin (IL)-1, IL-6, IL-8, and tumor necrosis factor (TNF)-α, and the subsequent massive recruitment and activation of neutrophils in the lower and upper airways. The inflammatory reactions are orchestrated by alveolar macrophages that carry specific endotoxin binding receptors [LPS binding protein, CD14, MD2, toll-like receptor (TLR) 4], which play a crucial role in the activation of these cells and the subsequent inflammatory processes (Gioannini et al. 2003). Considerable interindividual variability in response to endotoxin has been observed in endotoxin provocation experiments with naïve subjects (Kline et al. 1999).

Recent studies suggest that environmental endotoxin exposure might protect against the development of atopy and possibly allergic asthma (Gereda et al. 2000a, 2000b; Liu and Leung 2000; Martinez and Holt 1999; von Mutius et al. 2000). A low prevalence for atopy, hay fever, and to a lesser extent, asthma has been observed in the children and adolescents of farming families and first-year university students with a farming background (Braun-Fahrländer et al. 1999; Ernst and Cormier 2000; Kilpelaïnen et al. 2000; Portengen et al. 2002; Riedler et al. 2000; von Ehrenstein et al. 2000). Contact with livestock in the first year of life appeared to be one underlying determinant of this reduced risk. These observations are usually referred to as the “hygiene hypothesis.” Hence, exposure to endotoxin may reflect two sides of a coin: *a*) producing a protective effect with regard to atopy, and *b*) inducing inflammation that leads to nonallergic asthma. Two recent studies of adult farmers are in agreement with this hypothesis (Douwes et al. 2002). In the European Community Respiratory Health Survey, which investigates occupational asthma in 15,637 randomly selected people 20–44 years of age, the highest risk of asthma was shown for farmers [odds ratio (OR) = 2.6; 95% confidence interval (CI), 1.3–5.4] and agricultural workers (OR = 1.8; 95% CI, 1.0–3.2) (Kogevinas et al. 1999). An increased risk of asthma morbidity and mortality for farmers has been reported in several other studies as well (Fishwick et al. 1997; Neijari et al. 1996; Toren et al. 1991). Farmers involved in animal production seem to have the highest risk for asthma compared with subjects not involved in animal production (Melbostad et al. 1998). In the same study population, asthma in the absence of atopic sensitization to common allergens was associated with an increased exposure to endotoxin (Eduard et al. 2004). Other studies have reported conflicting results for the association of endotoxin exposure and asthma in farmers (Kimpbell-Dunn et al. 1999; Omland et al. 1999; Vogelzang et al. 1999). A study among Dutch pig farmers showed that the prevalence of atopic sensitization decreased sharply with increasing occupational endotoxin exposure, with the lowest prevalence at levels above 750 EU/m^3^ (Portengen et al. 2005). A study from Iowa showed an increased prevalence of childhood asthma on farms with increasing numbers of swine (Merchant et al. 2005). This raises the question as to whether exposures that begin in adulthood may lead to lowered risk for atopic sensitization as well, as opposed to the prevailing belief that a healthy worker selection is responsible for this phenomenon.

### Domestic endotoxin exposure and allergy and asthma in children

Most research on domestic endotoxin exposures has focused on the question of whether this exposure can explain the observations made in several studies describing a protective effect related to the development of atopy and allergic asthma in children who have grown up on small, traditional farms (Braun-Fahrländer et al. 1999; Klintberg et al. 2001; Portengen et al. 2002; Riedler et al. 2000; von Ehrenstein et al. 2000). It was indicated that contact with livestock reduced the risk of atopic asthma in children (von Ehrenstein et al. 2000) and young adolescents (Portengen et al. 2002). Although no specific protective factors were determined in these studies, it has been suggested that respiratory exposure to endotoxin may play an important role (Klintberg et al. 2001; von Ehrenstein et al. 2000), as it is well known, especially from occupational studies, that animal husbandry is associated with high exposures to bacterial endotoxin. However, exposures to other bioaerosol components such as fungi, gram-positive and gram-negative bacteria, bacterial DNA motifs, storage mites, and allergens from crops and animals are expected to be higher on farms and could also play a role.

The immune system is known to be skewed toward a proatopic direction during fetal and perinatal life. It has been proposed that proinflammatory microbial products, such as bacterial endotoxin, prokaryotic DNA, and glucans, markedly modulate the response of the immune system away from its tendency to develop atopic immune responses (Liu and Leung 2000; Martinez and Holt 1999). This may be a dose-dependent phenomenon, with low doses of these compounds providing some protective effects (as accounted for in the hygiene hypothesis of allergic asthma and atopy) and higher doses leading to a skewed and harmful response. Bacterial endotoxin and prokaryotic DNA can strongly induce IL-12 production by antigen-presenting cells, leading to the elaboration of interferon (IFN)-γ, IL-18, and other mediators. These mediators, many of which are transduced through one of the conserved TLR, are well recognized as promoting T helper (Th)1 (counter to Th2) responses. It has also been shown that the protective effect of farming exposure is regulated by a TLR2 response, as only children with the wild type of this gene were protected from allergy, given they were born on a farm (Eder et al. 2004). More recently, however, the promotion of “regulatory” responses (e.g., regulatory CD4^+^ T-cells and antigen-presenting cells such as dendritic cells and macrophages) has received prominent attention. These cells, when activated by microbial products referred to as pathogen-association molecular patterns (PAMPs) use IL-10 and TGF-β to mediate their attenuation effects; regulatory responses can downregulate both Th1 and Th2 immune responses. This category of inflammation may account for the observations that both Th1-mediated diseases (e.g., diabetes mellitus) and Th2-mediated diseases (asthma and atopic disorders) have been rising in industrialized countries over the past several decades where children are exposed to lower levels of microbial products and infections than in the past or in preindustrialized or agricultural societies. Further infections associated with eosinophilia (e.g., helminth infestations), that promote both Th2 and regulatory responses, can protect against asthma and atopy (Kline et al. 1998; Shirakawa, 1997; Yazdanbakhsh et al. 2002).

To date few studies have produced direct *in vivo* evidence that endotoxin exposure may protect against the development of atopy by enhancing Th1 responses. A U.S. study of infants with documented wheezing episodes showed that endotoxin levels were correlated with IFN-γ–producing T cells (Th1) but not with IL-4–, IL-5–, or IL-13–producing cell proportions (Th2) (Gereda et al. 2000b). A Swiss–German study of farm children showed a decreased capacity to release IFN-γ, TNF-α, IL-10, and IL-12 in peripheral blood leucocytes upon stimulation with LPS, with increasing endotoxin load in the beds (Braun-Fahrländer et al. 2002), which could be a consequence of “exhaustion” of the atopic immune system as proposed in association to atopy by Kruger et al. (2004). Animal experimental studies with ovalbumin and endotoxin have not yielded consistent results.

Studies that found a consistent protective effect of farming exposure against atopy (Braun-Fahrländer et al. 2002; von Mutius et al. 2000) have shown only a weak protective effect against asthma itself, or they have shown a dual response in children with atopic asthma and allergy to be lower with increasing LPS exposure, and contrary to this, an increased prevalence of nonatopic wheeze with increasing LPS exposure (Braun-Fahrländer et al. 1999). There is considerable evidence that endotoxin exposure may both exacerbate pre-existing asthma and induce new asthma. Several studies have also shown that endotoxin in house dust is associated with exacerbations of preexisting asthma in children and adults (Michel et al. 1991, 1996, 1997). A cohort study in 499 infants with a familial predisposition to asthma or allergy showed that early indoor endotoxin exposure was associated with an increased risk of repeated wheeze during the first year of life rather than a decreased risk (relative risk = 1.6; 95% CI, 1.03–2.38) (Shirakawa et al. 1997). A recent study of endotoxin in 831 homes across the United States demonstrated that indoor endotoxin was a significant risk factor for asthma symptoms, medication use, and wheezing. The adjusted OR for households with both bedding and bedroom floor endotoxin exceeding 19.6 EU/mg compared with those below was 2.83 (95% CI, 1.01–7.87). This effect was seen regardless of allergy status (Thorne et al. 2005).

Thus, for asthma alone, there is consistent evidence that endotoxin is both a secondary and primary cause of asthma, and that this occurs through nonatopic (i.e., nonimmunoglobulin E–mediated) mechanisms.

Because of the health effects related to environmental exposures, exposure standards have been suggested (Rylander 1997). The Health Council of the Netherlands proposed a health-based occupational exposure limit of 50 EU/m^3^ over 8 hr for the working environment, which has been modified to 200 EU/m^3^ because of feasibility issues. The introduction of this standard has been postponed because agricultural industries cannot meet this level, but exposure reduction action plans are being implemented so that this level can be met in a few years.

## Workshop Recommendations

### Priority research needs

- Candidate agents: Most research has been focused on specific gases, organic dust, or on bioaerosols containing endotoxins. Occupational studies indicate that exposures occur to other agents, such as antibiotics and disinfectants, and that these agents may be related to increased risks for respiratory disease.
- Odor: There is a need to investigate in greater detail psychophysiologic responses related to malodor exposures in people living in proximity to swine CAFOs, exploring different potential mechanisms. The influence of factors such as mood and coping styles on perceived responses to odors and physiologic responses has been minimally investigated in relation to CAFO exposures.
- CAFO mixed exposures: CAFO exposures involve, by definition, exposure to complex mixtures. These include pulmonary irritants, inflammatory agents, odoriferous compounds, allergens, and antibiotics. Problems of the interaction in mixtures need to be addressed.
- Environmental particulate matter exposures: Studies of particulate-matter exposure in rural areas are needed because of the huge gap in knowledge. Exposure mechanisms for particulates are expected to be different than those for gases because particulates from CAFOs are biologically active and are known to be relatively large. Therefore, sedimentation out of the air is expected to be considerable at short distances. Resuspension in the air, walk-in, and take-home exposures are important candidate mechanisms of transfer leading to exposure indoors. Finally, exposures arising from the handling and distribution of manure to the fields as primary aerosol and secondary re-suspension need to be addressed.
- Analytical techniques: International harmonization of methods for bioaerosol exposure assessment is needed. In the case of harmonization of endotoxin assay, the United States should give serious consideration to adoption of the existing European Committee for Standardization protocol.
- Susceptibility and genetics: Only scarce information is available for evaluating the susceptibility of groups to the effects of organic dust exposure. The candidate genes responsible for changes in the reactions to specific agents have been only sporadically investigated.
- Susceptibility and gender: Recent studies from Canada suggest that women are more prone than men to develop asthma from working in CAFOs. This points to the need for renewed investigations regarding gender effects in workers exposed to high concentrations of organic dust.
- Outcome assessment: There is a need to collect information regarding respiratory health in people living in proximity to CAFOs. Respiratory symptom status and lung function should be measured, as has been done in confinement workers in the last decades. Information on sensitization rates to common allergens and presence of allergic responses is crucial, as clear differences exist with nonrural populations and within rural populations, depending on early childhood exposure to animals. Recent developments in outcome assessment include more sensitive markers of inflammation, for example, IL-1β, IL-8, TNFα, C-reactive protein, and new sources to be studied by noninvasive approaches such as tear fluid, nasal lavage, exhaled breath condensate, and whole blood. These markers can elucidate the nature of the inflammatory response and facilitate a more detailed interpretation of the available information.
- Design: Panel studies have been shown to be powerful tools in air pollution research and should be pursued in studies of communities exposed to organic dust and CAFO emissions. They enroll sensitive individuals as a starting point and consider exacerbations of disease over time as the end point of interest. Exposure assessment in these studies requires combinations of exposure modeling, use of time-activity patterns and personal exposure assessment to calibrate the modelling. Alternatively, large-scale studies using hospital admission data in combination with spatial analysis to map the patients’ homes and schools to CAFOs may be a cost effective and useful additional approach.

### Translation of science to policy

- Surveillance studies of workers in agricultural industries and panel studies in communities are warranted.
- The livestock industry should promote good housekeeping practices locally and worldwide and develop hygienic strategies to reduce exposure in the workplace and emissions to the general environment. Occupational health and hygiene expertise is scarce in these industries and should be improved. The cost effectiveness of various technological solutions should be established.
- Exposure standards should be promulgated for some key agents, including endotoxin, and where they exist, exposure levels should be maintained below the current standards. International guidelines for occupational and community health are needed for specific toxicants.

## References

[b1-ehp0115-000298] Attwood P, Brouwer R, Ruigewaard P, Versloot P, de Wit R, Heederik D (1987). A study of the relationship between airborne contaminants and environmental factors in Dutch swine confinement buildings. Am Ind Hyg Assoc J.

[b2-ehp0115-000298] Avery R, Wing S, Marshall S, Schiffman S (2004). Odor from industrial hog farming operations and mucosal immune function in neighbors. Arch Environ Health.

[b3-ehp0115-000298] Braun-Fahrländer C, Gassner M, Grize L, Neu U, Sennhauser FH, Varonier HS (1999). Prevalence of hay fever and allergic sensitization in farmer’s children and their peers living in the same rural community. Clin Exp Allergy.

[b4-ehp0115-000298] Braun-Fahrländer CH, Riedler J, Herz U, Eder W, Waser M, Grize L (2002). Environmental exposure to endotoxin and its relation to asthma in school-age children. N Engl J Med.

[b5-ehp0115-000298] Brouwer R, Heederik D, van Swieten P (1990). IgG4 antibodies against pig-derived antigens. Am J Ind Med.

[b6-ehp0115-000298] Cormier Y, Boulet LP, Bedard G, Tremblay G (1991). Respiratory health of workers exposed to swine confinement buildings only or to both swine confinement buildings and dairy barns. Scand J Work Environ Health.

[b7-ehp0115-000298] Crook B, Robertson JF, Glass SA, Botheroyd EM, Lacey J, Topping MD (1991). Airborne dust, ammonia, microorganisms, and antigens in pig confinement houses and the respiratory health of exposed farm workers. Am Ind Hyg Assoc J.

[b8-ehp0115-000298] Donham KJ, Cumro D, Reynolds SJ, Merchant JA (2000). Dose-response relationships between occupational aerosol exposures and cross-shift declines of lung function in poultry workers: recommendations for exposure limits. J Occup Environ Med.

[b9-ehp0115-000298] Donham KJ, Zavala DC, Merchant JA (1984). Respiratory symptoms and lung function among workers in swine confinement buildings: a cross-sectional epidemiological study. Arch Environ Health.

[b10-ehp0115-000298] Douwes J, Doekes G, Heinrich J, Koch A, Bischof W, Brunekreef B (1998). Endotoxin and β(1→3)-glucan in house dust and the relation with home characteristics: a pilot study in 25 German houses. Indoor Air.

[b11-ehp0115-000298] Douwes J, Heederik D (1997). Epidemiologic investigations of endotoxins. Int J Occup Environ Health.

[b12-ehp0115-000298] Douwes J, Pearce N, Heederik D (2002). Does environmental endotoxin exposure prevent asthma?. Thorax.

[b13-ehp0115-000298] Douwes J, Zuidhof A, Doekes G, Van der Zee S, Wouters I, Boezen M (2000). β(1→3)-Glucan and endotoxin in house dust and peak flow variability in children. Am J Respir Crit Care Med.

[b14-ehp0115-000298] Eder W, Klimecki W, Yu L, von Mutius E, Riedler J, Braun-Fahrländer C (2004). Toll-like receptor 2 as a major gene for asthma in children of European farmers. J Allergy Clin Immunol.

[b15-ehp0115-000298] Eduard W, Douwes J, Omenaas E, Heederik D (2004). Do farming exposures cause or prevent asthma? Results from a study of adult Norwegian farmers. Thorax.

[b16-ehp0115-000298] Ernst P, Cormier Y (2000). Relative scarcity of asthma and atopy among rural adolescents raised on a farm. Am J Respir Crit Care.

[b17-ehp0115-000298] Fishwick D, Pearce N, D’Souza W, Town I, Armstrong R, Kogevinas M (1997). Occupational asthma in New Zealanders: a population based study. Occup Environ Med.

[b18-ehp0115-000298] Gereda JE, Leung DYM, Liu AH (2000a). Levels of environmental endotoxin and prevalence of atopic disease [Letter]. JAMA.

[b19-ehp0115-000298] Gereda JE, Leung DYM, Thatayatikom A, Price MR, Klinnert MD, Liu AH (2000b). Relation between house-dust endotoxin exposure, type 1 T-cell development, and allergen sensitisation in infants at high risk of asthma. Lancet.

[b20-ehp0115-000298] Gioannini TL, Teghanemt A, Zarember KA, Weiss JP (2003). Regulation of interactions of endotoxin with host cells. J Endotoxin Res.

[b21-ehp0115-000298] Harries MG, Cromwell O (1982). Occupational asthma caused by allergy to pigs’ urine. BMJ (Clin Res Ed).

[b22-ehp0115-000298] Jagielo PJ, Thorne PS, Watt JL, Frees KL, Quinn TJ, Schwartz DA (1996). Grain dust and endotoxin inhalation challenges produce similar inflammatory responses in normal subjects. Chest.

[b23-ehp0115-000298] Katila ML, Mantyjarvi RA, Ojanen TH (1981). Sensitisation against environmental antigens and respiratory symptoms in swine workers. Br J Ind Med.

[b24-ehp0115-000298] Keikhaefer MS, Donham KJ, Whitten P, Thorne PS (1995). Cross seasonal studies of airborne microbial populations and environment in swine buildings: implications for worker and animal health. Ann Agric Environ Med.

[b25-ehp0115-000298] Kilpelaïnen M, Terho O, Helenius H, Koskenvuo M (2000). Farm environment in childhood prevents the development of allergies. Clin Exp Allergy.

[b26-ehp0115-000298] Kimpbell-Dunn M, Bradshaw L, Slater T, Erkinjuntti-Pekkanen R, Fishwick D, Pearce N (1999). Asthma and allergy in New Zealand farmers. Am J Ind Med.

[b27-ehp0115-000298] Kline JN, Cowden JD, Hunninghake GW, Schutte BC, Watt JL, Wohlford-Lenane CL (1999). Variable airway responsiveness to inhaled lipopolysaccharide. Am J Respir Crit Care Med.

[b28-ehp0115-000298] Kline JN, Waldschmidt TJ, Businga TR, Lemish JE, Weinstock JV, Thorne PS (1998). Modulation of airway inflammation by CpG oligodeoxynucleotides in a murine model of asthma. J Immunol.

[b29-ehp0115-000298] Klintberg B, Berglund N, Lilja G, Wickman M, van Hage-Hamsten M (2001). Fewer allergic respiratory disorders among farmers’ children in a closed birth cohort from Sweden. Eur Respir J.

[b30-ehp0115-000298] Kogevinas M, Anto JM, Sunyer J, Tobia A, Kromhout H, Burney P (1999). Occupational asthma in Europe and other industrialised areas: a population-based study. Lancet.

[b31-ehp0115-000298] Kullman GJ, Thorne PS, Waldron PF, Marx JJ, Ault B, Lewis DM (1998). Organic dust exposures from work in dairy barns. Am Ind Hyg Assoc J.

[b32-ehp0115-000298] Kruger T, Sigsgaard T, Bonefeld-Jorgensen EC (2004). Ex vivo induction of cytokines by mold components in whole blood of atopic and non-atopic volunteers. Cytokine.

[b33-ehp0115-000298] Liu AH, Leung YM (2000). Modulating the early allergic response with endotoxin. Clin Exp Allergy.

[b34-ehp0115-000298] Martinez FD, Holt PG (1999). Role of microbial burden in aetiology of allergy and asthma. Lancet.

[b35-ehp0115-000298] Melbostad E, Eduard W, Magnus P (1998). Determinants of asthma in a farming population. Scand J Work Environ Health.

[b36-ehp0115-000298] Merchant JA, Naleway AL, Svendsen ER, Kelly KM, Burmeister LF, Stromquist AM (2005). Asthma and farm exposures in a cohort of rural Iowa children. Environ Health Perspect.

[b37-ehp0115-000298] Michel O, Ginanni R, Duchateau J, Vertongen F, Le Bon B, Sergeysels R (1991). Domestic endotoxin exposure and clinical severity of asthma. Clin Exp Allergy.

[b38-ehp0115-000298] Michel O, Kips J, Duchateau J, Vertongen F, Robert L, Collet H (1996). Severity of asthma is related to endotoxin in house dust. Am J Respir Crit Care Med.

[b39-ehp0115-000298] Michel O, Nagy AM, Schroeven M, Duchateau J, Nève J, Fondu P (1997). Dose-response relationship to inhaled endotoxin in normal subjects. Am J Respir Crit Care Med.

[b40-ehp0115-000298] Neijari C, Tessier JF, Letenneur L, Dartigues JF, Barberger-Gateau P, Salamon R (1996). Prevalence of self-reported asthma symptoms in a French elderly sample. Respir Med.

[b41-ehp0115-000298] Omland O, Sigsgaard T, Hjort C, Pedersen OF, Miller MR (1999). Lung status in young Danish rurals: the effect of farming exposure on asthma-like symptoms and lung function. Eur Respir J.

[b42-ehp0115-000298] Peterson RD, Wicklund PE, Good RA (1964). Endotoxin activity of a house dust extract. Allergy.

[b43-ehp0115-000298] Portengen L, Preller L, Tielen M, Doekes G, Heederik D (2005). Endotoxin exposure and atopic sensitization in adult pig farmers. J Allergy Clin Immunol.

[b44-ehp0115-000298] Portengen L, Sigsgaard T, Omland O, Hjort C, Heederik D, Doekes G (2002). Low prevalence of atopy in young Danish farmers and farming students born and raised on a farm. Clin Exp Allergy.

[b45-ehp0115-000298] Radon K Schulze A van Strien R Ehrenstein V Praml G Nowak D 2005. Respiratory health and allergy among young adults from rural areas of Lower Saxony [in German]. In: Proceedings of 50th Annual Meeting of the German Society for Medical Informatics, Biometrics and Epidemiology, 15 September 2005, Freiburg, Germany. Available: http://www.egms.de/en/meetings/gmds2005/05gmds237.shtml [accessed 7 November 2005].

[b46-ehp0115-000298] Riedler J, Eder W, Oberfeld G, Schreuer M (2000). Austrian children living on a farm have less hay fever, asthma and allergic sensitization. Clin Exp Allergy.

[b47-ehp0115-000298] Rylander R (1997). Evaluation of the risks of endotoxin exposure. Int J Occup Environ Health.

[b48-ehp0115-000298] Rylander R, Donham KJ, Hjort C, Brouwer R, Heederik D (1989). Effects of exposure to dust in swine confinement buildings—a working group report. Scand J Work Environ Health.

[b49-ehp0115-000298] Sandström T, Bjermer L, Rylander R (1992). Lipopolysaccaride (LPS) inhalation in healthy subjects increases neutrophils, lymphocytes and fibronectin levels in bronchoalveolar lavage fluid. Eur Respir J.

[b50-ehp0115-000298] Schenker M, Christiani D, Cormier Y, Dimich-Ward H, Doekes G, Dosman J (1998). Respiratory health hazards in agriculture. Am J Respir Crit Care Med.

[b51-ehp0115-000298] Shirakawa T, Enomoto T, Shimazu S, Hopkin JM (1997). The inverse association between tuberculin responses and atopic disorder. Science.

[b52-ehp0115-000298] Thorne PS, Kulhánková K, Yin M, Cohn R, Arbes SJ, Zeldin DC (2005). Endotoxin Exposure is a risk factor for asthma: the national survey of endotoxin in U.S. housing. Am J Respir Crit Care Med.

[b53-ehp0115-000298] Thorne PS, Kulhankova K, Yin M, Cohn R, Arbes S, Zeldin DC (2003). Endotoxin in house dust is associated with asthma but not allergy [Abstract]. Am J Respir Crit Care Med.

[b54-ehp0115-000298] Toren K, Horte L-G, Jarvholm B (1991). Occupation and smoking adjusted mortality due to asthma among Swedish men. Br J Ind Med.

[b55-ehp0115-000298] Vogelzang PF, van der Gulden JW, Folgering H, Kolk JJ, Heederik D, Preller L (1998). Endotoxin exposure as a major determinant of lung function decline in pig farmers. Am J Respir Crit Care Med.

[b56-ehp0115-000298] Vogelzang PF, van der Gulden JW, Tielen MJ, Folgering H, van Schayck CP (1999). Health-based selection for asthma, but not for chronic bronchitis, in pig farmers: an evidence-based hypothesis. Eur Respir J.

[b57-ehp0115-000298] von Ehrenstein OS, von Mutius E, Illi S, Baumann L, Böhm O, von Kries R (2000). Reduced risk of hay fever and asthma among children of farmers. Clin Exp Allergy.

[b58-ehp0115-000298] Von Essen S, Romberger D (2003). The respiratory inflammatory response to the swine confinement building environment: the adaptation to respiratory exposures in the chronically exposed worker. J Agric Safety Health.

[b59-ehp0115-000298] von Mutius E, Braun-Fahrländer C, Schierl R, Riedler J, Ehlermann S, Maisch S (2000). Exposure to endotoxin or other bacterial components might protect against the development of atopy. Clin Exp Allergy.

[b60-ehp0115-000298] Yazdanbakhsh M, Kremsner PG, van Ree R (2002). Allergy, parasites, and the hygiene hypothesis. Science.

[b61-ehp0115-000298] Zhiping W, Malmberg P, Larsson BM, Larsson K, Larsson L, Saraf A (1996). Exposure to bacteria in swine-house dust and acute inflammatory reactions in humans. Am J Respir Crit Care Med.

